# A novel ecological momentary assessment app for the investigation of daily cognitive functioning in breast cancer survivors: a feasibility study

**DOI:** 10.1007/s00520-025-09470-1

**Published:** 2025-04-29

**Authors:** Annalee L. Cobden, Jake Burnett, Jacqueline B. Saward, Alex Burmester, Mervyn Singh, Juan F Domínguez D, Priscilla Gates, Jocelyn Lippey, Karen Caeyenberghs

**Affiliations:** 1 Cognitive Neuroscience Unit, School of Psychology, Deakin University, Geelong, Australia; 2 St Vincent’s Hospital, Melbourne, Australia; 3 Alberta Children’s Hospital Research Institute, The University of Calgary, Calgary, AB Canada; 4 Cumming School of Medicine, Department of Radiology, University of Calgary, Calgary, AB Canada; 5 Peter MacCallum Cancer Centre, Melbourne, Australia; 6 Department of Breast Surgery, St Vincent’s Hospital Melbourne, Fitzroy, VIC Australia; 7 Department of Surgery, University of Melbourne, Melbourne, Australia; 8 Neuroplasticity and Multimodal Imaging (NMI) Lab, Cognitive Neuroscience Unit, School of Psychology, Deakin University, Melbourne Burwood Campus, 221 Burwood Highway, Burwood, VIC 3125 Australia

**Keywords:** Breast cancer, Cancer-related cognitive impairment, Cognition, Psycho-oncology, Ecological momentary assessment

## Abstract

**Purpose:**

Breast cancer survivors often experience cancer-related cognitive impairment (CRCI), such as problems with memory and attention. However, typical neuropsychological test batteries are unable to capture the day-to-day variability of cognition and may be underestimating CRCI. The present study aims to assess the feasibility, usability, and validity of a novel ecological momentary assessment (EMA) app of cognition.

**Methods:**

Nineteen breast cancer survivors 6–36-month post-chemotherapy and 28 healthy controls completed the NIH Toolbox Cognition Battery. Subsequently, participants completed the EMA app (once a day, for 30 days) comprising four cognitive tasks assessing processing speed, working memory, inhibition, and attention. At the conclusion of the app, participants completed a usability questionnaire on which content analysis was performed. Feasibility was assessed against eight criteria, including accessibility, app compliance, and technical smoothness. Convergent construct validity was assessed using Spearman’s correlation analyses between the NIH toolbox and the EMA app.

**Results:**

Five of eight feasibility criteria were met, including accessibility, app motivation, participation rate, drop-out, and data collection. Additionally, our content analyses revealed four themes important to usability: self-development, altruism, engagement, and functionality. Majority of the EMA tasks were moderately positively correlated with the corresponding constructs of the NIH toolbox tasks (R’s range 0.55–0.64), indicating better performance on the EMA app coincided with better performance on the NIH toolbox.

**Conclusions:**

Our findings show the app was accessible, participants were motivated to complete sessions, and our tasks showed good construct validity.

**Implications for Cancer Survivors:**

Our novel EMA app can be used as a comprehensive cognitive measure in cancer survivors.

**Supplementary Information:**

The online version contains supplementary material available at 10.1007/s00520-025-09470-1.

Approximately up to 60% of breast cancer survivors report experiencing cancer-related cognitive impairments (CRCI), including attentional problems, reduced processing speed, and working memory deficits [1–4]. However, meta-analysis of the literature indicates that prevalence varies depending on method of assessment [3] and time since treatment [1]. Survivors frequently report experiencing cognitive difficulties at a rate of 44%, which is notably higher than the 21–34% prevalence of deficits identified through neuropsychological assessments [3]. Longitudinal work shows progression of cognitive function varies from person-to-person with some survivors (32%) experiencing the greatest cognitive difficulty prior treatment and improving at 1-year post-treatment, while approximately 27% decline in cognitive ability 1-year post-treatment [1]. Overall neuropsychological assessment estimate around 21% of breast cancers survivors experience CRCI 2–3-year post-chemotherapy treatment [3]. CRCI can have far-reaching impacts on daily life [5, 6] and for a subset of survivors can last years after completion of cancer treatment [7].

Previous studies on CRCI utilised neuropsychological assessments as objective assessments of cognitive functioning. However, they are typically conducted in a single behavioural testing session, which only provides a brief snapshot of cognition. The incongruity between retrospective self-reported and objective cognitive performance may be due to several factors such as the involvement of psychological distress, discrepancy of definitions of impairment, and/or compensatory processes [8, 9]. Psychological distress quantified as anxiety, depressive symptoms, and fatigue are consistently found to be associated with CRCI and often a significant predictor of self-reported cognitive dysfunction [10, 11]. However, the gap between self-reported and neuropsychological assessments of CRCI has not consistently been fully explained by psychological distress. Therefore, compensatory processes may explain the inconsistency of CRCI as they are more easily enlisted while performing tasks in a quiet lab environment than in environments with everyday distractions [12]. This implies the summary measures of the neuropsychological test batteries typically employed provide an incomplete representation of how cognitive symptoms are experienced.

Ecological momentary assessment (EMA) provides the opportunity to repeatedly measure and track cognitive symptoms within a person’s natural environment over days to weeks [13]. EMAs overcome the limitations of previous studies that utilise single point neuropsychological assessments in laboratory settings by minimising recall bias, maximising ecological validity, and allowing the study of day-to-day changes in behaviour in real-world contexts. EMA has been widely used to assess cognition in a range of clinical populations, including neurological and psychiatric disorders [14–16]. EMA presents inherent challenges such as participant reactivity to self-monitoring through daily assessments, standardisation of procedures, and the added participant burden of completing daily tasks [13]. Therefore, establishing feasibility, usability, and validity of EMA cognitive tasks is essential. A systematic review of 12 cognitive EMA studies, in primarily healthy older adults, generally reported high construct validity of their EMA cognitive tasks [16]. The included EMA studies had sampling periods of 1–14 days and typically assessed one to three cognitive constructs (e.g. working memory and attention). All but one study supplied participants with a study specific mobile device or personal digital assistant. These results suggest that ambulatory neuropsychological assessments are assessing the same constructs; however, they can provide unique insight into day-to-day cognitive function.

There are a growing number of EMA studies in cancer populations [17]. A scoping review of 12 EMA studies in people with cancer reported that previous studies had low attrition rates (range 1–25%) and low missing data (range 1–39%), indicating EMA is feasible and promising in cancer survivors [17]. For example, one study examined diurnal mood and pain in breast cancer survivors showing increases in negative mood when they experienced fatigue and pain [18]. However, all of the included EMA studies examined emotional wellbeing and mental health. According to our knowledge, only two studies (of the same sample) have investigated CRCI using EMA in breast cancer survivors [19, 20]. These studies used ambulatory variations of the digit symbol task, dot memory, and an n-back task to assess processing speed, episodic memory, and attention respectively. The authors used three level (prompt, day, and person) multilevel models to assess variability of cognitive scores within and between participants, and overall variance of scores on three cognitive tasks was attributable to 54–83% of within-subject variability. This suggests that within-person fluctuations explained over half of the variance of results, which is not accounted for by traditional single session testing and highlights the need for EMA to adequately capture it. However, the study did not have a control group to compare variability. The research group also found poor daily self-reported cognitive performance coincided with higher fatigue levels (assessed using a visual analogue scale) [20]. These studies demonstrate the potential of EMA cognitive tasks in elucidating the day-to-day experience in cognitive functioning of breast cancer survivors.

In the current study, we will present a novel EMA app (called MindTrax), which is able to capture day-to-day, intra-individual variability of cognitive functioning. Compared with previous EMA studies, our app is conducted on participants own devices and assesses a wider array of cognitive functions. The included cognitive functions are processing speed, executive working memory, spatial working memory, and inhibition/attention as recommended by the International Cognition and Cancer Task Force [21]. Moreover, we will administer this EMA app in two groups (i.e. breast cancer survivors and healthy controls), over 30 days. Our aims are threefold: firstly, we will evaluate the feasibility and usability of our novel app, and study design and procedures within each group and across groups. Secondly, we will further enrich our understanding of the user experience of the app by performing thematic content analysis. Finally, we will test the convergent construct validity of the cognitive tasks of the novel app by performing correlation analyses between NIH Toolbox and EMA tasks.

## Methods

### Participants

The breast cancer survivors were recruited via oncology clinics (Victorian Breast and Oncology Care in East Melbourne (JL)) and non-profit organisations (including Breast Cancer Awareness Australia and ThinkPink). Breast cancer participants were eligible if they met the following criteria: (a) 6–36-month post-completion of chemotherapy; (b) completed > four cycles of chemotherapy; (c) no documented metastatic disease; (d) if taking hormonal therapy, they had been taking the therapy for > 3 months; (e) in cancer remission. In the present study, we focus on breast cancer survivors 6–36-month post-chemotherapy, rather than acute, to allow stabilisation of cognitive outcomes. In line with previous studies (e.g. Small and colleagues [19]), we also focus on survivors who have received at least a standard dose of chemotherapy (i.e. four cycles) for consistency of the sample.

The healthy controls were recruited through convenience sampling via social media (e.g. Facebook community pages), word-of-mouth, and flyer advertisements (e.g. noticeboards). Healthy controls had to have no history of cancer to meet the inclusion criteria. Inclusion criteria for both groups were as follows: (a) daily access to a smartphone (android or iOS) with internet; (b) aged 18–65 years; (c) understood English and were able to provide written informed consent. Further exclusion criteria for both groups were as follows: (a) history of neurological or major psychiatric disorder (such as uncontrolled schizophrenia, active suicidality/self-harm); (b) a history of intravenous drug abuse; (c) a concussion within the past 12 months; (d) pregnancy at the time of the study (as this study is part of a larger project that involved magnetic resonance imaging, and pregnancy is a contraindication for this) (see study schema in supplementary A).

The study was approved by Deakin Human Research Ethics (2020–404 and 2021–129) and informed written consent was obtained from all participants. Participants were reimbursed for their time with a voucher.

### Data collection procedures

#### In-person cognitive test battery

All 47 participants completed the NIH Toolbox for Cognition (www.nihtoolbox.org) Version 2 [22] on a supplied iPad Air 2 (246-mm screen) in a quiet testing room (approximately 30–min completion time). The NIH toolbox for cognition is a standardised test battery which has previously been used in breast cancer survivors [23, 24] as well as the broader cancer and non-cancer literature [23, 25, 26]. Reaction time (milliseconds) was extracted from the flanker task, dimensional change card sorting task, and pattern comparison sorting speed test. In addition, the flanker reaction time ratio was calculated by dividing reaction time of the congruent trials by reaction time of the incongruent trials. Lastly, the picture sequence memory test score (percentage correct responses) was extracted.

#### IQ measure

Participants completed the National Adult Reading Test (NART) to obtain an estimate of full-scale intelligence quotient (IQ) [27]. The NART full-scale IQ estimate has been found to have a strong correlation with the Wechsler Adult Intelligence Scale-IV [28].

#### Ecological momentary assessment-cognitive tasks

**Fig. 1:**
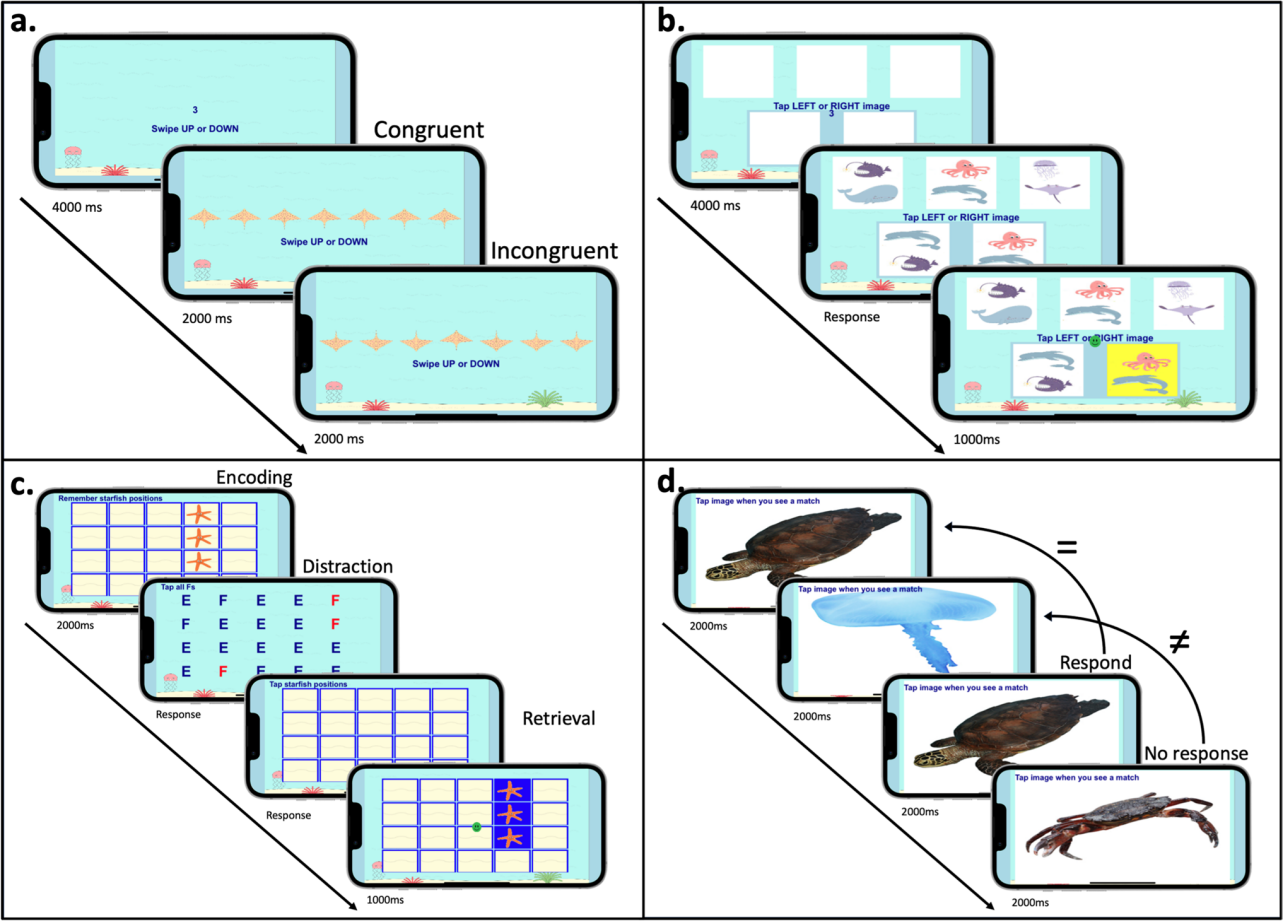
Overview of the MindTrax cognitive tasks: **a** flanker stingray task, **b** flanker stingray task, **c** starfish memory task, **d** 2-back task

The MindTrax app included four cognitive tasks and a set of questions assessing emotional wellbeing. In the present study, we will focus on the data of the cognitive tasks. The app was available to download for free on both Android (Google Play Store) and iPhone (App Store) mobile smartphones (interested readers can contact the senior author to test the app). Participants were asked to complete the MindTrax app once a day at a fixed schedule at 8:00 pm for 30 days. Each session took approximately 5 min. An automatic text reminder was sent to participants at 8:30 pm, if they had not completed the app. The order of these four tests was randomised each day of testing within each participant. The MindTrax app included four brief cognitive tasks: (a) card matching task; (b) flanker task; (c) starfish memory task; (d) 2-back task (Fig. 1, full specifications see supplementary B). The tasks assess processing speed, inhibition/attention, spatial working memory, and executive working memory respectively.

#### Usability questionnaire

At the completion of the EMA testing, participants were emailed a usability questionnaire adapted from previous studies [29, 30]. Participants were asked to respond to 17 statements on a 5-point Likert scale from 1—strongly disagree through to 5—strongly agree. The statements fell into five subscales (see supplementary C): (1) Subjective experience; (2) Ease of use; (3) Technical smoothness; (4) Understanding; (5) Methodological acceptability. The usability questionnaire included five free response questions used for the inductive content analysis (e.g. What did you enjoy the most, and why).

### Data analysis

#### Feasibility analysis

To evaluate the feasibility of our novel MindTrax app, we defined eight feasibility criteria (see Table 1), based on previous studies [31, 32]. Specifically, we identified four feasibility outcome measures and their criteria for success related to the smartphone app (accessibility, app compliance, technical smoothness, and app motivation) and four criteria concerning study design and procedures (participation rates, drop-out, usability assessment time period, and data collection). We set stringent success criteria for the different feasibility criteria.

#### Usability

Usability was assessed by calculating the descriptive statistics (such as mean, range) for each subscale of the usability questionnaire.

#### Inductive content analysis of usability

To evaluate the usability of the app, we adopted an inductive content approach to analysis of the open-ended responses on the usability questionnaire [33, 34]. This technique allowed capturing of themes and patterns associated with subjective perceptions to inform clinical understanding in small sample sizes [34]. Inductive content analysis was conducted across the groups to ensure data saturation in developing the usability themes. The information was analysed in three phases: open coding, creating categories, and abstraction, consistent with previous literature (extended methods supplementary D) [35]. In this study, trustworthiness of the qualitative content analysis was maintained through co-coding (PG) [35, 36]. Coding and analysis were discussed with the wider research team to clarify and interpret emerging themes and subthemes [33].

#### Convergent construct validity analysis

We tested the convergent construct validity of the cognitive tasks of the EMA app by performing Spearman’s correlation (due to skewed data) between the variables of the EMA app and the NIH toolbox across all participants. Construct validity was performed with participant groups combined to maintain statistical power. Correlations of interest were as follows: (a) Attention (EMA flanker RT and NIH flanker RT); (b) Inhibition (EMA flanker RT ratio and NIH flanker ratio); (c) Processing speed (EMA card matching RT and NIH pattern comparison sorting speed RT); (d) Spatial working memory (EMA starfish memory RT and NIH dimensional change sorting RT); (e) Executive working memory (2-back accuracy and picture sequence memory score). Consistent with the literature, the criterion for convergent construct validity was set at *r* ≥ 0.5 [37].

Finally, to rule out practice effects of the repeated-assessments, paired samples *t*-tests were performed on all the outcome variables of the EMA tasks between participants second completed session and last session (adjusted for multiple comparisons using the false discovery rate, FDR < 0.05). Participants second session was used (rather than the first session) to allow for participants to familiarise themselves with the EMA tasks. These *t*-tests and correlation analyses were conducted using R (4.2.0) [38].

## Results

### Participants

A total of 47 participants (mean = 42.91 years, SD = 13.58) were recruited, including 19 breast cancer survivors (mean = 51.93 years, SD = 9.31, 19 = female) and 28 healthy controls (mean = 36.79 years, SD = 12.54, 18 females). The breast cancer survivors significantly differed from healthy controls with regards to age (*t*(45) = 4.45, *p* < 0.001) and gender (*X*^2^ (45) = 12.16, *p* < 0.001). There were no significant differences between the two groups for full-scale IQ (*t*(45) = 1.61, *p* = 0.115) and level of education was similar between the groups.

Breast cancer survivors were on average 18-month post-chemotherapy (SD = 7.78) and the most common combination of chemotherapy agents breast cancer survivors underwent were doxorubicin and cyclophosphamide, and paclitaxel (6/19). All participants underwent surgery as part of their treatment and a majority of them also received radiation therapy (15/19), and ongoing hormonal treatment (11/19). Ten of the breast cancer survivors reported currently receiving professional mental health support. A detailed overview of the clinical and demographic characteristics of the breast cancer survivors can be found in supplementary E.

All participants (46) completed the app, and one of the breast cancer participants was removed from the correlational analysis because they used an iPad not a phone (due to size differences between tablet and phone screens, which can affect cognitive performance). Participants completed an average of 78.84% of sessions (range 20–100% of sessions). A total of 39 participants completed the usability questionnaire and their responses were used to assess feasibility and usability. Responses provided by 36 participants (18/19 BCS, 18/28 HC) were used in the inductive content analysis.

### Feasibility outcomes

Across groups, five out of the eight feasibility criteria were met, including accessibility, app motivation, participation rate, drop-out, and data collection (see supplementary material F). Specifically, 100% of participants reported they understood the app tasks; 85% of participants scored their experience as *somewhat agree* or above; 100% of participants who consented participated in the app; 82.98% completed the usability questionnaire; and 99.56% of started sessions of the cognitive tasks had complete data. App compliance, technical smoothness, and assessment time scale were not met: 78.84% of total possible sessions were completed; only 30.7% of participants reported experiencing no technical difficulties; and the usability questionnaire was completed on average 30.95 days after completing EMA tasks. Breast cancer survivors and healthy controls showed similar trends on most feasibility criteria (see table 1). However, breast cancer survivors did meet the criteria for success in app compliance: completing 82.11% of total possible sessions while healthy controls completed 76.54% and did not meet the criteria for success. Healthy controls reported better technical smoothness (38% no technical difficulties compared to 22.2% technical difficulties in the breast cancer survivor group) and were less likely to complete the usability questionnaire (Table 1).

**Table 1:** Feasibility criteria and results of app and study design in breast cancer survivors and healthy controls

Feasibility measure	Feasibility question	Criteria for success	Outcome			Success?
			Breast cancer survivors	Success?	Controls	
MindTrax						
Accessibility	Did participants understand the app tasks and questions?	All participants understood the tasks and question	94% (17/18) somewhat agreed or strongly agreed on understanding of the app. No participants strongly disagreed that they understood the app	Yes	100% (21/21) somewhat agreed or strongly agreed on understanding of the app	Yes
App compliance	Did participants complete all daily assessments across the 30 days?	Overall, participants complete an average of at least 80% of the daily assessments	82.11% of sessions (17–30) were completed	Yes	76.54% of sessions (6–30) were completed	No
Technical smoothness	Were participants able to complete assessments without technical difficulty?	Participants were able to complete 100% of attempted assessments	22.2% (4/18) strongly agreed, 16.7% (3/18) somewhat agree that no technical difficulties prevented them from completing any tasks	No	38% (8/21) strongly agreed, 14.3% (3/21) somewhat agree that no technical difficulties prevented them from completing any tasks	No
App motivation	Were participants motivated to complete the app sessions?	As assessed using the usability questionnaire, 80% of participants had at least a neutral impression of the app	83.3% (15/18) of participants chose somewhat agree or higher when asked if they enjoyed the overall experience of using the app	Yes	85.7% (18/21) of participants chose somewhat agree or higher when asked if they enjoyed the overall experience of using the app	Yes
Study design						
Participation rate	Did participants who agreed to take part in the study participate in the study?	At least 80% of participants who initially agreed to participate, participated	100% of participants who completed the consent form participated in the study	Yes	100% of participants who completed the consent form participated in the study	Yes
Drop-out	Did participants complete the MindTrax usability questionnaire?	At least 80% of participants complete the MindTrax usability questionnaire	95% (18 of 19) completed the questionnaire	Yes	75% (21 of 28) completed the questionnaire	Yes
Usability assessment period	Was the MindTrax usability questionnaire completed less than 7 days after the end of app sessions?	All participants who complete the MindTrax usability questionnaire, complete it within 7 days	4 of 18 participants completed it within 7 days	No	13 of 21 participants completed it within 7 days	No
Data collection	Was all the data collected without technical issues?	At least 90% of the data is collected without technical complications on the back-end server	2 of 1872 data points were missing	Yes	16 of 2480 data points were missing	Yes

### Usability outcomes

**Fig. 2:**
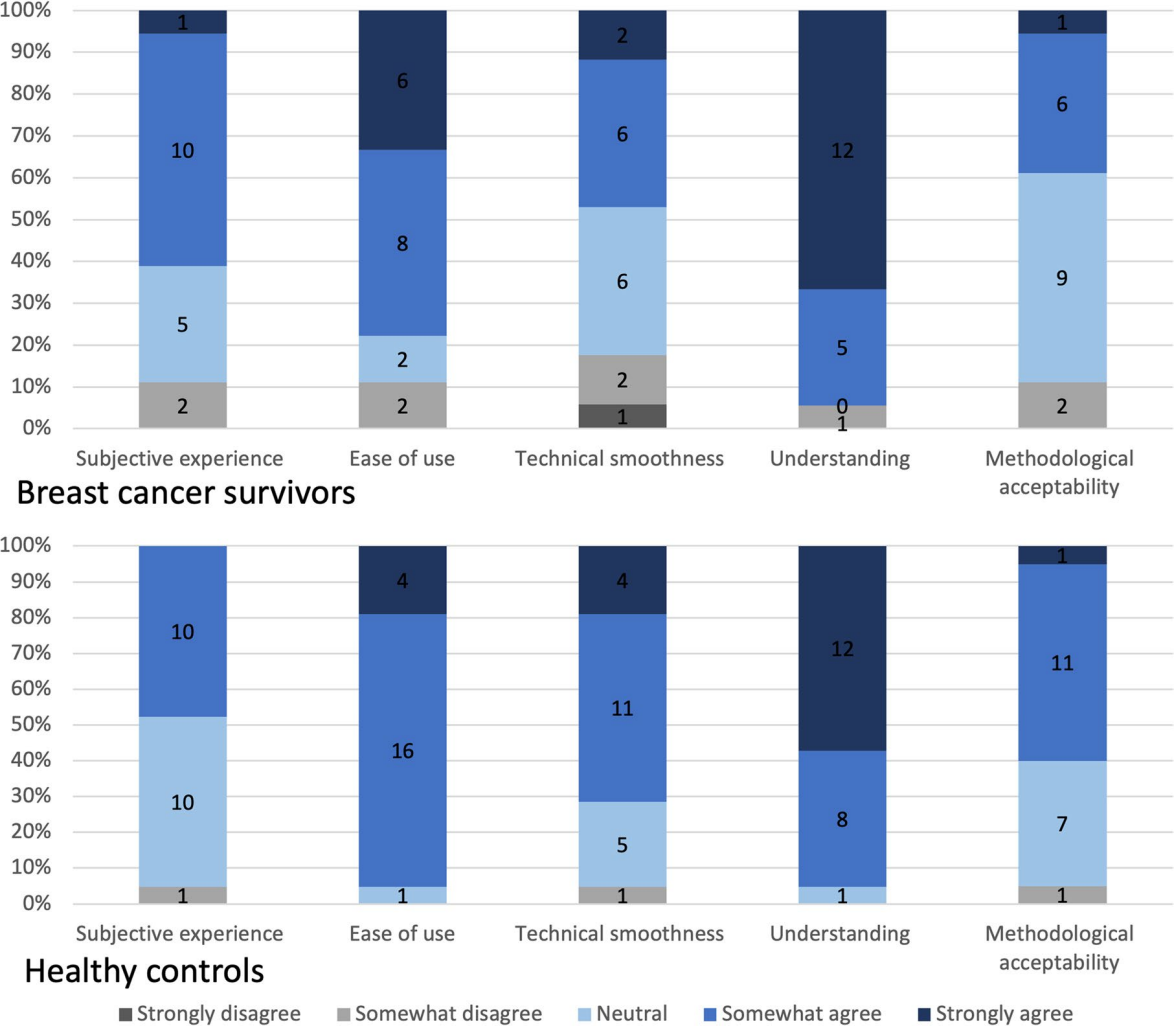
Scores on the five subscales of the MindTrax usability questionnaire in the breast cancer survivor group (top) and the healthy control group (bottom)

The usability questionnaire showed neutral to high agreement scores across the five subscales and similar results across groups (Fig. 2). The subscale of overall understanding the app had the highest scores with a mean score of 4.58, SD = 0.77, range = 2–5 for survivors and 4.57, SD = 0.53, range = 3.5–5 for healthy controls. Across the groups, the majority (37/39) of participants *strongly agreed/somewhat agreed* on understanding questions. The subscales of ease of use and overall subjective experience obtained mean scores of 4.14 (SD = 0.90, range = 2–5) and 3.69 (SD = 0.65, range = 2.5–5) respectively for survivors. While controls had mean scores of 4.4 (SD = 0.41, range 3.5–5) for ease of use and 4.17 (SD = 0.53, range 2.8–5) for technical smoothness. Finally, breast cancer survivors scored the subscales for methodological acceptability and technical smoothness of using the app were lower with a mean score of 3.68 (SD = 0.69, range = 2–5) and 3.66 (SD = 0.89, range = 1.4–5). Healthy controls two lowest subscales were subjective experience (mean = 3.82, SD = 0.45, range = 2.75–5) and methodological acceptability (mean = 3.87, SD = 0.76, range = 1.75–5). Overall, methodological acceptability had the lowest number of participants who chose *strongly agree/agreed* (19/39) while 4/39 people chose *strongly disagree/somewhat disagree*. Combined group outcomes for usability showed the similar trends as the group data and can be accessed in supplementary material G.

### Inductive content analysis of MindTrax usability

Four themes were generated from the open responses on the usability questionnaire: (1) self-development; (2) altruism; (3) engagement; (4) functionality (see supplementary H and I).

Theme 1: Self-development.

Participants reported enjoying the challenge of the cognitive tasks on the app. Specifically, participants enjoyed the challenge of the 2-back task and trying to improve their performance each day:

               "Seeing how well I could or could not remember tasks" (BC08).

However, participants, particularly breast cancer survivors, reported feelings of self-judgment, when asked what they enjoyed least in the day. Some participants enjoyed the open-ended questions as it gave them the opportunity to reflect on their mood each day while some participants highlighted that they did not like being prompted to reflect on the worst part of their day.

Theme 2: Altruism.

People reported feeling positive about being given the opportunity to be part of a research study and help others:

"The fact that I was helping with research" (HC02)

Theme 3: Engagement.

Participants reported finding the app enjoyable. Specifically, participants recalled enjoying the ocean theme and that the tasks felt like games:

               "The memory game was fun. Reminded me of the old childhood game" (HC20).

While some participants found the sound design affected enjoyment of the app. Moreover, several participants reported the timing of the tasks difficult, and due to competing practical issues, such as social engagements and work commitments. Given this, many reported forgetting to complete the tasks.

Theme 4: Functionality.

Participants reported some of the functionality of the app prevented them from completing some sessions. Of particular concern for participants, were no notifications, and timing and consistency of notifications.

               "The text reminders came at random times" (HC04).

### Convergent construct validity: correlation analysis

Descriptive statistics of the dependent variables of the NIH toolbox and the EMA app tasks can be found in supplementary J. There were no significant improvements of the outcome variables of the EMA app tasks from the second to the last session (see supplementary K and L) apart from reaction time of the card-matching task. In other words, there were no training effects on the cognitive tasks of the EMA app over the sampling period of 30 days. We only observed a training effect for the reaction time of the EMA card matching task for healthy controls (*t*(27) = 4.70, *p* < 0.001) and breast cancer survivors (*t*(17) = 4.57, *p* < 0.01).

**Fig. 3:**
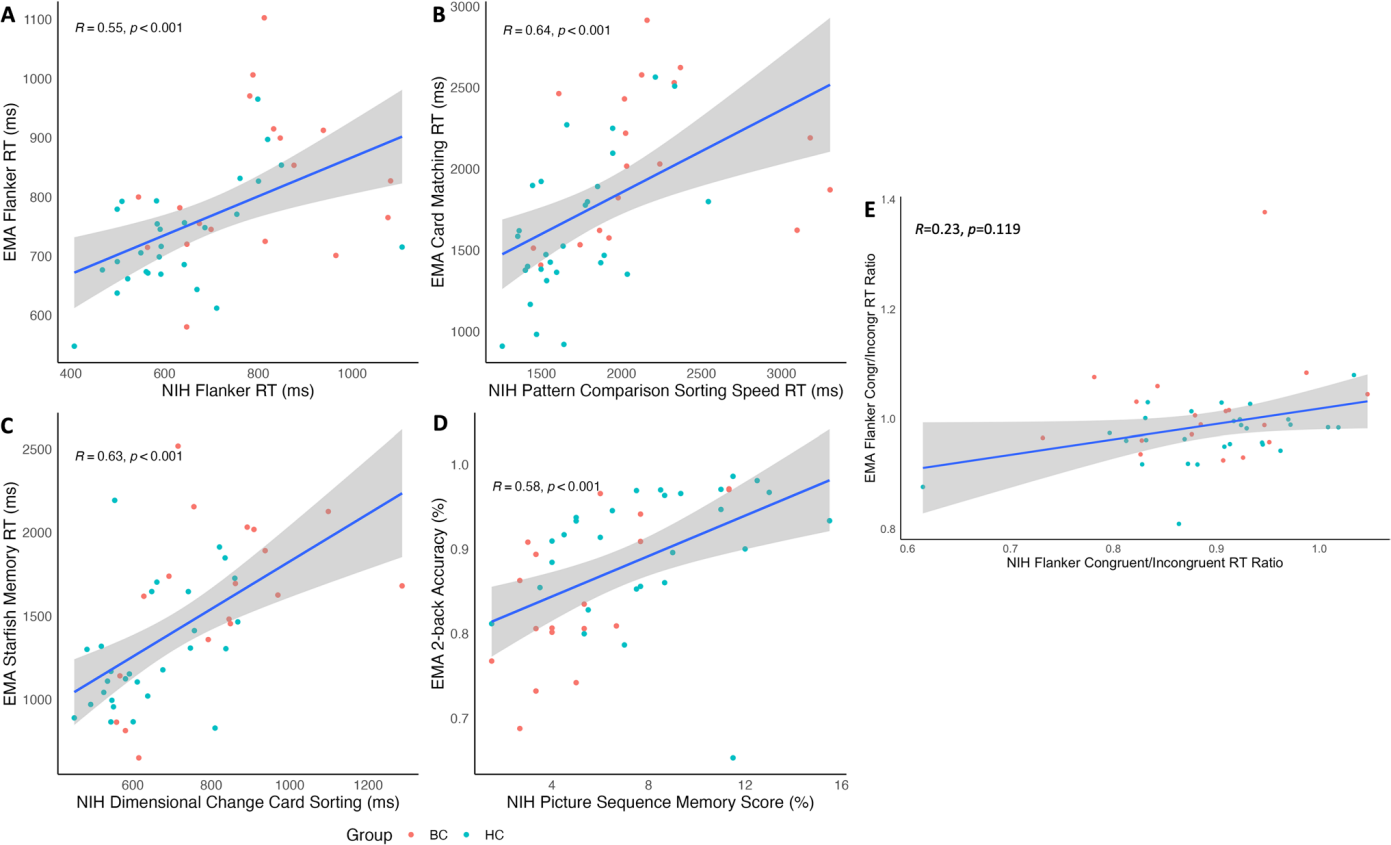
Spearman’s correlations between EMA cognitive task-dependent variables and NIH cognitive task-dependent variables

Spearman’s correlation analyses revealed significant moderate positive correlations between the majority of the EMA cognitive tasks and the NIH toolbox tasks (Fig. 3 and supplementary M). Specifically, we observed positive moderate relationships between EMA flanker RT and NIH flanker RT(*r*[45] = 0.55, *p* < 0.001); EMA card matching RT and NIH pattern comparison sorting speed (*r*[45] = 0.64, *p* < 0.001); EMA starfish memory RT and dimensional change card sorting RT (*r*[45] = 0.63, *p* < 0.001); and EMA 2-back accuracy and NIH picture sequence score (*r*[45] = 0.58, *p* < 0.001). In other words, better performance on the EMA cognitive tasks coincided with better performance on the NIH toolbox. However, we did not find a significant relationship between EMA flanker RT ratio and NIH flanker RT ratio (*r*[45] = 0.23, *p* = 0.12).

## Discussion

Our study suggests our novel EMA app MindTrax is a feasible method to obtain measures of cognitive functioning in breast cancer survivors and healthy adults. Specifically, our results show that a majority of feasibility criteria were met. Furthermore, MindTrax demonstrated usability (with self-development, altruism, engagement, and functionality as themes) and high convergent construct validity.

Previous EMA research has demonstrated EMA designs are feasible in people with cancer [17, 19]. However, the feasibility of ambulatory cognitive assessments has not been established in both breast cancer survivors and a healthy control group. While our results showed five of the eight feasibility criteria were met, criteria for app compliance, technical smoothness, and usability assessment period were not attained. App compliance (78.84%) was close to attainment (80%) and is consistent with the rate of compliance in prior cognitive task-based EMA studies. A previous meta-analysis of mobile cognitive EMA studies found a mean response rate of 79.2% (58–87%) [16]. Moreover, group-specific compliance rates revealed breast cancer participants did meet the criteria for app compliance (82.11%), consistent with a previous cognitive EMA study in breast cancer survivors [20]. This is particularly encouraging as our sampling period was longer than previous cognitive EMA studies [16, 17], demonstrating the potential for being able to track CRCI in breast cancer survivors over longer time periods.

Regarding technical smoothness, variations in the operating systems of participants’ devices introduced technical complications (such as issues with notifications), which prevented some participants from completing sessions. Though, EMA studies frequently report missing data often due to missed sessions [16, 17]. In particular, EMA studies in people with cancer have found up to 39% of their data missing [17]. Future studies should employ advanced mixed models to deal with the missing data [39]. The final unmet feasibility criterion, participants completing usability questionnaire within 7 days, may be explained by the usability questionnaire being sent via email. Future studies should integrate the usability questionnaire in the final session of the app.

Unlike the present study, few studies have employed self-report measures and open-ended opinions on usability for cognitive EMA tasks to explore participants’ experience. We found participants’ overall experience of using the app was rated highly, with few participants reporting negative experiences with the app. Our inductive content analysis revealed that while some participants enjoyed the self-reflection and challenge aspects of completing the tasks, some breast cancer survivors felt self-judgment when they felt they performed poorly on a task. Our results are consistent with previous cognitive EMA studies in people with mild cognitive impairments who reported mixed responses when asked if being more aware of their memory was unpleasant [30]. Future studies should be mindful that feelings of self-judgment may arise in clinical groups when completing cognitive EMA. Moreover, feelings of self-judgment of breast cancer survivors with and without CRCI as should be further explored. Participants highlighted that they found notifications to be an important factor in completing daily sessions consistently. Ensuring consistent notifications should be a priority in future EMA studies. Several participants highlighted that they enjoyed being a part of research and the potential for the research to help others. Similar altruistic sentiments were highlighted in a recent study exploring the motivation to participate in CRCI research on patients with newly diagnosed aggressive lymphoma [33].

Importantly, our EMA tasks were correlated with in-person neuropsychological assessments (average *r* > 0.53), consistent with previous cognitive EMA tasks [16, 30, 40]. This suggests the MindTrax tasks have good convergent construct validity. However, the EMA flanker task congruent/incongruent reaction time ratio did not correlate with the equivalent NIH flanker ratio. This may have been due to several methodological factors such as a smaller condition effect in the ambulatory task, too easy (due to the salience of the stimuli) [41] and ceiling effects. Ease of the task can be addressed by adjusting the salience of the stimuli and/or decreasing the time the stimulus is presented. Our tasks also showed moderate-strong associations with NIH tasks of unrelated constructs. However, it is not uncommon for cognitive assessments to be associated with assessments of other constructs, for example several NIH cognitive toolbox tasks show moderate correlations with other NIH tasks [42]. There was evidence of training effects in our card matching task reaction time due to ceiling effects of the EMA card matching task. All other EMA variables did not show evidence for training effects, supporting the feasibility of EMA cognitive tasks over longer durations of time.

### Study limitations

While our study demonstrates the feasibility, usability, and validity of our novel EMA app tasks, there were several limitations. Firstly, due to our relatively small sample size and our groups not being well matched with gender and age, we analysed the construct validity of the cognitive tasks as one group. Despite not being well matched on gender and age, our groups were well matched on education and IQ which are confounding factors. The gender and age differences are however important to consider when looking at our group results for feasibility and usability. We recommend future large-scale studies should be undertaken to comprehensively describe EMA cognitive assessments. Secondly, our app required internet access while completing tasks, which may have negatively impacted participants’ engagement in the tasks; however, our participants did not highlight this as a barrier to completing tasks. Finally, while we have adapted previously validated cognitive tasks, there is potential for bias in our testing and reporting due to developing MindTrax ourselves.

### Clinical implications

Our findings revealed that a long sampling period, i.e. daily sampling of cognitive functioning across 30 days, is feasible in breast cancer survivors. This finding suggests that our novel EMA app may enable the investigation of daily cognitive deficits throughout the different stages of cancer treatment and survivorship. Furthermore, the breast cancer survivors (and non-cancer controls) both rated the experience of using the app positively, supporting the usage of cognitive assessments in an app format. The app also enables the engagement of patients in rural or remote communities, thus broadening its geographical scope, scale, and reach. App-based cognitive assessments will enhance equity in healthcare delivery in future work.

## Conclusion

CRCI can have a profound effect on daily life functioning in breast cancer survivors. However, the single session neuropsychological assessments do not fully capture the symptom experience reported by survivors. Our MindTrax results revealed that the app and the study design was feasible, and it demonstrated reliable and valid estimates of cognitive performance between the EMA cognitive tasks and the corresponding constructs on the well-established NIH toolbox. Finally, participants also reported the app was acceptable and usable, with positive comments. However, the feelings of self-judgment and technical issues that some participants experienced should be important design considerations in future work using mobile implementations of cognitive EMA. Future work will test this novel EMA app in large-scale training studies to track cognitive functioning with cognitive training.

## Supplementary Information

Below is the link to the electronic supplementary material.

ESM 1 (PDF 656 KB)

## Data Availability

The data that support the findings of this study are available on request from the corresponding author. The data are not publicly available due to privacy or ethical restrictions.
