# Supplementary material for: A novel ecological momentary assessment app for the investigation of daily cognitive functioning in breast cancer survivors: a feasibility study
Source: Support Care Cancer. 2025 Apr 29;33(5):434. doi: 10.1007/s00520-025-09470-1 (PMC12041178; doi:10.1007/s00520-025-09470-1)
Supplement: Supplementary file 1 — (PDF 656 KB) [file 520_2025_9470_MOESM1_ESM.pdf]

Supplementary Material A

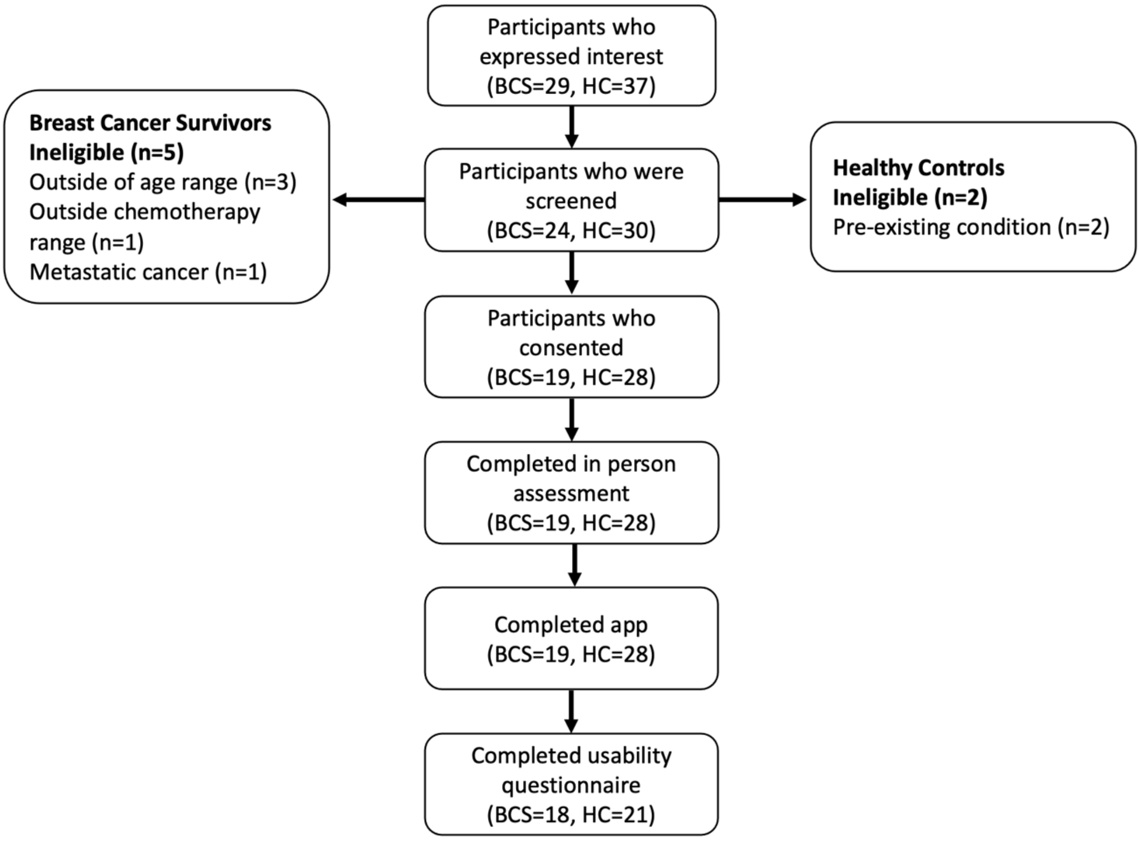

Study schema

Supplementary Material B

Expanded methods: MindTrax cognitive task specifications.

Feedback was provided after each trial, in the form of a red frowning face (incorrect) or a green smiling face (correct).

(i) The flanker stingray's task is a variant of the Flanker task, which assesses inhibitory control and selective attention.[1] Preceding each stimulus was a 4000ms count down presented as 'get ready', '3', '2', '1' each displayed for 1000ms. The stimulus comprised seven stingrays (see Figure 1A) in a row, with a stimulus duration of 2000 ms. Participants were instructed to swipe the screen in the direction of the centre stingray as fast and as accurate as possible. In the congruent condition, the six flanking stingrays were positioned in the same direction as the central stingray. In the incongruent condition, the flanking stimuli were positioned in the opposing direction of the centre stingray. Participants completed 10 trials per session. Reaction time (RT) was computed as an average across trials of each session. Besides RT, the flanker reaction time ratio was calculated as congruent reaction time/incongruent RT.

(ii) The card matching task is an adaptation of the pattern comparison task and measures processing speed.[2] As shown in Figure 1B, the stimulus consisted of five cards, three in a row on the upper half of the screen, and two cards on the lower half of the screen. Participants were instructed to match one of the lower cards with one of the top cards as fast and as accurate as possible. The stimulus was presented until the participant responded. Each stimulus was preceded by a 4000ms count down, 'get ready', '3', '2', '1,' which were all displayed for 1000ms each. The interstimulus interval was 1000ms. The location of the correct stimuli was randomly determined . Participants completed 10 trials per session and average RT was calculated for each session.

(iii) The starfish memory task is a form of the symmetry span task and assesses spatial working memory.[3] Participants were required to remember the spatial locations of three starfish randomly placed in a (4 x 5) grid for 2000ms, as displayed in Figure 1C. After the distraction task (tapping on the F's amongst E's), the participants needed to reproduce the positions of the three starfish. Participants completed three

of these trials each session. The interstimulus interval time was 1000ms. The dependent variables were average reaction time and the accuracy scores.

(iv) The 2-back task (an implementation of the n-back paradigm) assesses executive working memory, and has been previously employed in ambulatory assessments. [4,5] The participants are required to tap on the image if the image is the same as 2 trials prior (thus 2-back). Each image was presented for 2000ms (Figure 1D). Participants completed 15 trials per session. Average reaction time and accuracy of responses were calculated for each session.

References

1. Eriksen BA, Eriksen CW. Effects of noise letters upon the identification of a target letter in a nonsearch task. Percept Psychophys. 1974 Jan;16(1):143–9.
2. Carlozzi NE, Beaumont JL, Tulsky DS, Gershon RC. The NIH Toolbox Pattern Comparison Processing Speed Test: Normative Data. Archives of Clinical Neuropsychology. 2015 Aug;30(5):359–68.
3. Kane MJ, Hambrick DZ, Tuholski SW, Wilhelm O, Payne TW, Engle RW. The Generality of Working Memory Capacity: A Latent-Variable Approach to Verbal and Visuospatial Memory Span and Reasoning. J Exp Psychol Gen. 2004 Jun;133(2):189–217.
4. Kirchner WK. Age differences in short-term retention of rapidly changing information. J Exp Psychol. 1958;55(4):352–8.
5. Sliwinski MJ, Mogle JA, Hyun J, Munoz E, Smyth JM, Lipton RB. Reliability and Validity of Ambulatory Cognitive Assessments. Assessment. 2018 Jan 15;25(1):14–30.

Supplementary Material C

Note: MindTrax was previously known as Daily Cognition and Mood (DCaM)

Usability Questionnaire

The following usability questionnaire is based on mobile ecological momentary assessment usability questionnaires used in previous studies (Bartels 2020, Spook, Kok, Van Empelen 2013). There are 17 statements which fall into five main domains; 1) Subjective experience, 2) Ease of use 3) Technical smoothness, 4) Understanding, 5) Methodological acceptability. Responses are answered on a 5-point Likert scale from 1- strongly disagree through to 5- strongly agree. There are also 5 open-ended questions about using the app. The test will be administered and formatted using the Research Electronic Data Capture (REDcap) system.

The statements for each category are as follows:

1) Subjective experience
   1. I enjoyed the overall experience of using the DCaM app
   2. Using the app felt like a chore
   3. Completing the daily DCaM session interfered with my daily activities
   4. I was able to find the time to use the app each day

2) Ease of use
   5. Overall, the app was easy to use
   6. I think most people would be able to understand how to use the app quickly

3) Technical smoothness
   7. I could read the instructions and cues of the tasks in the app easily
   8. The app ran smoothly
   9. The questions displayed well on my phone
   10. The cognitive tasks displayed well on my phone
   11. There were no technical difficulties which prevented me from completing a session

3) Understanding
   12. It was clear what I needed to do for the cognitive tasks
   13. I understood what the questions were asking

5) Methodological acceptability
   14. I found the DCaM app instructions given by the researchers easy to follow
   15. I usually had my phone with me when I received the notification
   16. The notification was enough of a reminder to do the DCaM sessions
   17. The study was too long

Scoring: Items 2, 3, 17 will need to be reverse coded when reporting

References:

Bartels, S.L., Van Knippenberg, R.J.M., Malinowsky, C., Verhey, F.R.J., De Vugt, M.E., 2020. Smartphone-Based Experience Sampling in People With Mild Cognitive Impairment: Feasibility and Usability Study. JMIR Aging.. doi:10.2196/19852

Spook, J.E., Paulussen, T., Kok, G., Van Empelen, P., 2013. Monitoring Dietary Intake and Physical Activity Electronically: Feasibility, Usability, and Ecological Validity of a Mobile-Based Ecological Momentary Assessment Tool. Journal of Medical Internet Research.. doi:10.2196/jmir.2617

DCaM Usability Questionnaire

This questionnaire is designed to understand your experience using the Daily Cognition and Mood or DCaM app throughout your time participating in the study. All answers will be anonymous.

Please rate how much you agree with the following statements about your experience using the app. 1=strongly disagree, 2= somewhat disagree. 3=neutral, 4= somewhat agree, 5-strongly agree.

1. The notification was enough of a reminder to do the DCaM sessions
2. The app ran smoothly
3. I found the DCaM app instructions given by the researchers easy to follow
4. Using the app felt like a chore
5. The study was too long
6. I could read the instructions and cues of the tasks in the app easily
7. Overall, the app was easy to use
8. I usually had my phone with me when I received the notification
9. I think most people would be able to understand how to use the app quickly
10. The app ran smoothly
11. It was clear what I needed to do for the cognitive tasks
12. I enjoyed the overall experience of using the DCaM app
13. I was able to find the time to use the app each day
14. The questions displayed well on my phone
15. The notification was enough of a reminder to do the DCaM sessions
16. Completing the daily DCaM session interfered with my daily activities
17. There were no technical difficulties which prevented me from completing a session

The following questions about using the app can be answered with as much or as little detail as you would like.

1. What did you enjoy the most, and why?

2. What did you enjoy least, and why?

3. Did you have any technological difficulties? If so what were they?

4. If you didn’t complete one or more sessions, why?

5. Do you have any other suggestions or comments?

Supplementary Material D

Expanded methods: thematic content analysis.

The first step, open coding, consisted of data familiarisation by reading each open-ended response, making notes while reading and identifying key phrases and words to freely generate categories. The second step, creating categories, entailed the iterative development, organization, and reduction of the number of categories by grouping codes based on similarities and into broader categories. Data saturation was considered when there was replication in categories, confirming completeness and comprehensives of the data and in turn, facilitating abstraction. The final step, abstraction, involved the organization of the broader categories into salient themes and the respective descriptions.

Supplementary Material E

Overview of the demographic and clinical variables of our participants

Variable		BCS (19)	HC (28)
Sex			
	Female	19	18
	Male	0	10
Age			
	Mean (SD)	51.93 (9.31)	36.79 (12.54)
	Range	34.88-65.94	21- 61.99
Education			
	High School	3	5
	Bachelor	9	18
	Masters	6	4
	Doctorate	1	1
Estimated FSIQ			
	Mean (SD)	114.8 (3.82)	116.82 (4.68)
	Range	106.42-123.02	105.59-123.02
Clinical characteristics		**19**	
Chemotherapy			
	**Time since chemotherapy (months)**		
	Mean (SD)	18 (7.78)	
	Range	7-30	
	**Chemotherapy agents**		
	Doxorubicin, Cyclophosphamide,	3	
	Doxorubicin, Cyclophosphamide,, paclitaxel	8	
	Doxorubicin, Cyclophosphamide,, Paclitaxel & Carboplatin	1	
	Docetaxel and cyclophosphamide	3	
	Docetaxel and carboplatin	1	
	Not reported	3	
Surgery		19	
	**Surgery Type**		
	Lumpectomy	9	
	Mastectomy	11	
	Axillary dissection	5	
	**Surgery timing**		
	Before chemotherapy	7	
	After chemotherapy	9	
	Both	2	
Radiation			
	Yes	16	

No		3
Hormonal treatment		**11**
Tamoxifen		3
Letrozole		1
Arimidex		3
Anastrozole		2
Aromasin		1
Herceptin		1
Mental health support	10 (yes)	No (8)

Note: AC= FSIQ= full scale IQ

Supplementary Material F: Feasibility criteria and results of app and study design

			Outcome	
**Feasibility Measure**	**Feasibility question**	**Criteria for success**	**Overall**	**Success?**
**MindTrax**				
**Accessibility**	Did participants understand the app tasks and questions?	All participants understood the tasks and question.	97% (38/39) participants somewhat agreed or strongly agreed on understanding of the app. No participants reported they strongly disagreed that they understood the app.	Yes
**App compliance**	Did participants complete all daily assessments across the 30 days?	Overall, participants complete an average of at least 80% of the daily assessments.	78.84% of sessions were completed.	No
**Technical smoothness**	Were participants able to complete assessments without technical difficulty?	Participants were able to complete 100% of attempted assessments.	30.7% (12/39) strongly agreed that no technical difficulties prevented them from completing any tasks, 15.4% (6/29) somewhat agree.	No
**App motivation**	Were participants motivated to complete the app sessions?	As assessed using the usability questionnaire, 80% of participants had at least a neutral impression of the app.	85% (33/39) of participants chose somewhat agree or higher when asked if they enjoyed the overall experience of using the app.	Yes
**Study Design**				
**Participation rate**	Did participants who agreed to take part in the study participate in the study?	At least 80% of participants who initially agreed to participate, participated.	100% of participants who completed the consent form participated in the study.	Yes
**Drop-out**	Did participants complete the MindTrax usability questionnaire?	At least 80% of participants complete the MindTrax usability questionnaire.	82.98% of participants completed the usability questionnaire.	Yes
**Usability assessment period**	Was the MindTrax usability questionnaire completed less than 7 days after the end of app sessions?	All participants who complete the MindTrax usability questionnaire, complete it within 7 days.	17 of the 39 participants who completed the usability questionnaire, completed it within 7 days of app completion.	No
**Data collection**	Was all the data collected without technical issues?	At least 90% of the data is collected without technical complications on the back-end server.	99.56% of data was successfully reported, 19 of 4352 data points were missing.	Yes

Supplementary Material G

The usability questionnaire showed neutral to high agreement scores across the five subscales (Figure 2). The subscale of overall understanding the app had the highest scores with a mean score of 4.58, SD = 0.64, range = 2-5 and the majority (37/39) of participants *strongly agreed/somewhat agreed* on understanding questions. The subscales of ease of use and technical smoothness obtained mean scores of 4.28 (SD = 0.69, range = 2-5) and 3.94 (SD = 0.79, range = 1.4-5) respectively. Finally, the subscale scores for methodological acceptability and overall subjective experience of using the app scored lower with a mean score of 3.78 (SD = 0.73, range = 1.75-5) and 3.87 (SD = 0.55, range = 2.5-5). Specifically, methodological acceptability had the lowest number of participants who chose *strongly agree/agreed* (19/39) while 4/39 people chose *strongly disagree/somewhat disagree*.

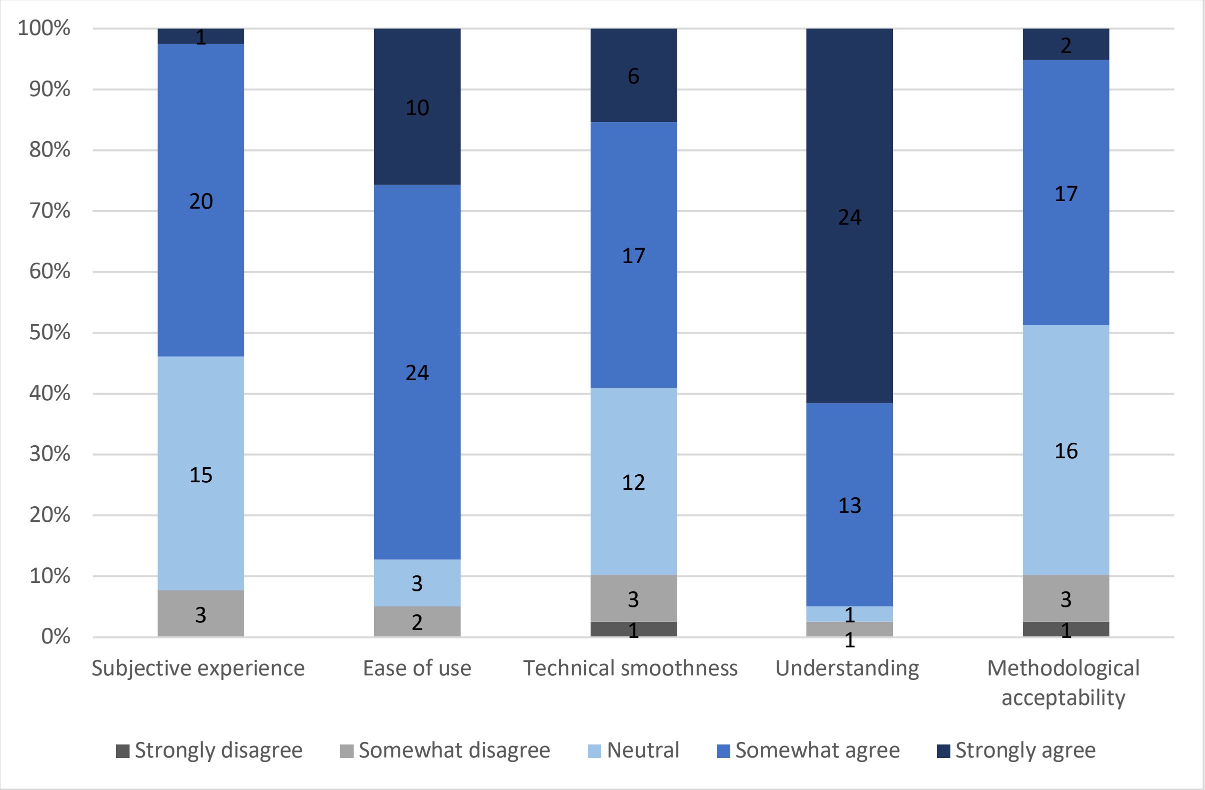

Supplementary Material H

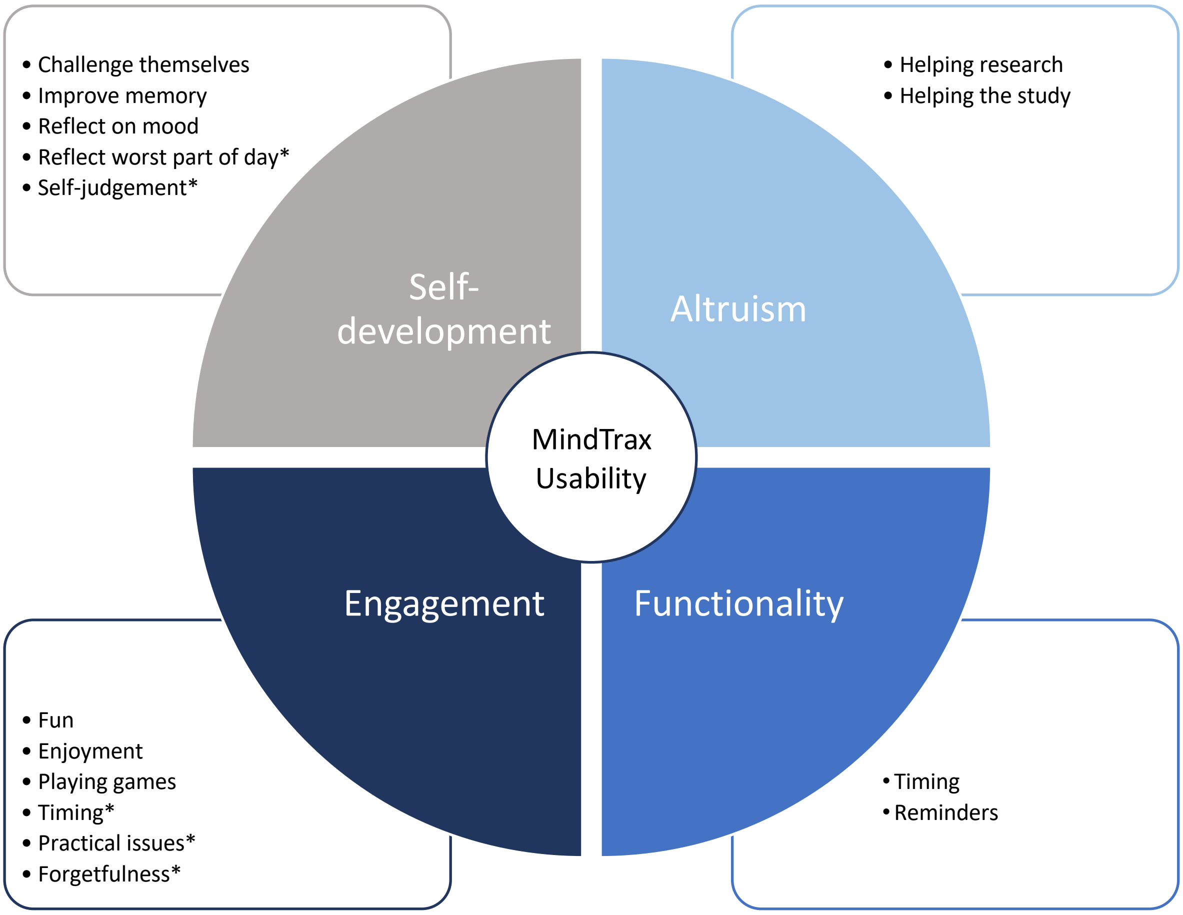

Themes elucidated through inductive content analysis of open-ended MindTrax usability questions. Asterix denote aspects which reduced the usability of MindTrax.

Supplementary Material I

Participant quotes that reflect study themes.

Theme	Quote
**Self-development**	*Failing a test! I don't like to fail…(BC06)*
	I wouldn't necessarily normally do this but it was interesting to consider how my mood was affected by what's going on in my life (HC18)
	I don't like to focus on negative aspects of the day (BC04)
**Altruism**	*Being a part of a study"(BC07).*
**Engagement**	*The 'ding' sound became a bit annoying after a while. (B09)*
	I was out with others and then forgot when I got home" (HC04)
**Functionality**	*Some notifications didn't work (HC20).*

Supplementary Material J

Descriptive statistics of NIH and EMA variables

Variable	Min	Max	Median	Mean	SD
NIH flanker score	0.95	1.00	1.00	1.00	0.01
NIH flanker RT	407.45	1106.23	658.20	697.05	168.54
NIH flanker RT ratio	0.62	1.05	0.91	0.89	0.08
EMA flanker RT (ms)	547.80	1102.71	751.32	767.33	113.70
EMA flanker ACC	0.78	1.00	0.99	0.98	0.04
EMA flanker RT ratio	0.81	1.38	0.99	0.99	0.08
EMA flanker second RT (ms)	0.00	1309.90	791.95	798.85	231.29
EMA flanker last RT (ms)	486.30	1160.80	753.60	784.80	148.40
EMA flanker second ACC	0.00	1.00	1.00	0.95	0.19
EMA flanker last ACC	0.80	1.00	1.00	0.99	0.03
NIH pattern comparison sorting speed score	0.97	1.00	1.00	0.99	0.01
NIH pattern comparison sorting speed RT (ms)	1255.48	3303.17	1819.26	1878.20	472.56
EMA card matching ACC	0.90	1.00	0.97	0.97	0.03
EMA card matching RT (ms)	908.95	2914.38	1699.74	1791.74	490.34
EMA card matching Second RT (ms)	898.30	4189.90	2073.95	2141.24	704.77
EMA card matching last RT (ms)	575.00	2820.70	1539.20	1651.73	503.72
EMA card matching second ACC	0.70	1.00	1.00	0.95	0.08
EMA card matching last ACC	0.90	1.00	1.00	0.98	0.04
NIH picture sequence memory score	1.50	15.50	6.25	6.89	3.42
NIH picture sequence memory RT (ms)	39218.01	140315.90	56668.53	65003.82	23852.52
EMA 2-back ACC	0.65	0.99	0.90	0.88	0.08
EMA 2-back RT	535.81	981.66	705.07	706.22	102.09
EMA2-back second RT (ms)	215.86	1012.40	722.38	703.58	182.62
EMA 2-back last RT (ms)	471.00	1072.43	654.98	693.05	155.38
EMA 2-back second ACC	0.14	1.00	0.89	0.82	0.23
EMA 2-back last ACC	0.43	1.00	1.00	0.90	0.14
NIH dimensional change card sorting score	0.87	1.00	1.00	0.98	0.03
NIH dimensional change card sorting RT (ms)	450.61	1286.41	668.74	708.20	175.71
EMA starfish memory RT (ms)	650.67	2517.46	1338.91	1411.40	443.37
EMA starfish memory ACC	0.12	0.91	0.67	0.60	0.22
EMA starfish memory second RT (ms)	664.33	4731.67	1575.33	1690.31	849.56
EMA starfish memory last RT (ms)	546.33	6304.33	1217.33	1449.28	975.66
EMA starfish memory second ACC	0.00	1.00	0.67	0.57	0.30
EMA starfish memory last ACC	0.00	1.00	0.67	0.63	0.36

Supplementary Material K

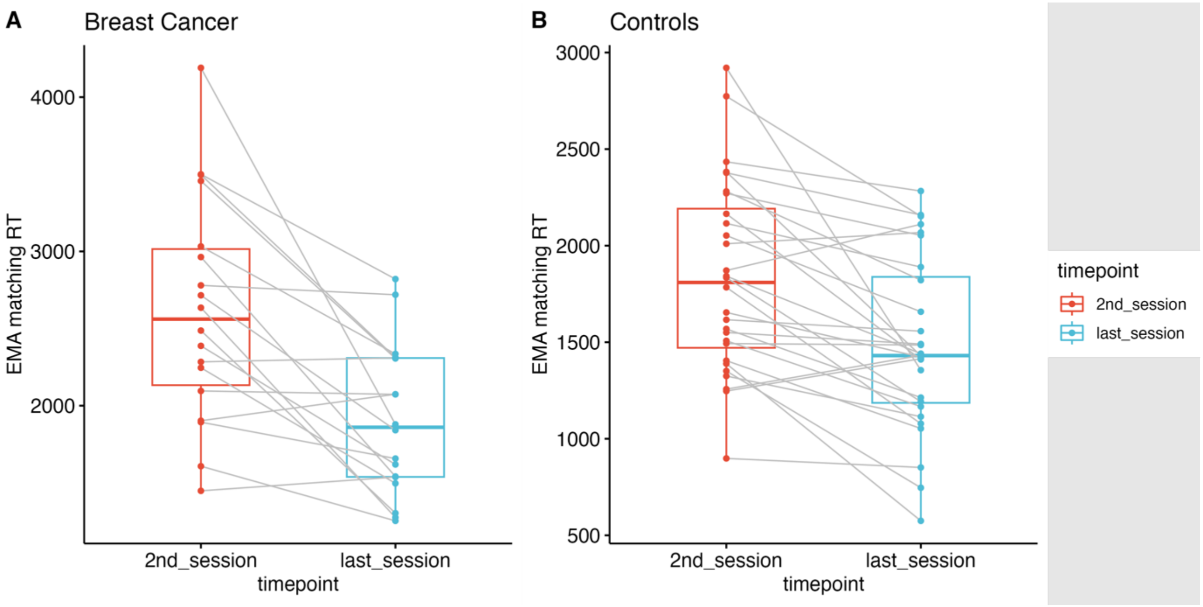

Boxplots of significant t-tests of card matching RT in both groups.

Supplementary Material L

Results of t-test between participants 2nd and final completions of EMA tasks.

	Healthy Controls			Breast Cancer Survivors		
Task	**t-statistic**	**df**	**p.adj**	**t-statistic**	**df**	**p.adj**
Reaction time						
Flanker	0.26	27	0.87	0.45	17	0.66
Card matching	4.70	27	0.00054***	4.57	17	0.0022**
2-back	2.37	27	0.1	-1.47	17	0.25
Starfish memory	-0.16	27	0.87	2.63	17	0.070
Accuracy						
Flanker	-1.44	27	0.32	-0.99	17	0.38
Card matching	-1.77	27	0.23	-1.76	17	0.19
2-back	-0.70	27	0.79	-2.21	17	0.11
Starfish memory	-0.49	27	0.84	-1.37	17	0.25

Note: *** =p < .001, **=p < .01

Supplementary Material M

Spearman's correlation analysis results between EMA and NIH cognitive tasks.

NIH	EMA				
	**Flanker RT**	**Flanker RT Ratio**	**Card matching RT**	**Starfish memory RT**	**2-back ACC**
Flanker RT	0.55***	0.20	0.63***	0.45**	-0.42**
Flanker RT ratio	0.04	0.23	-0.08	0.16	0.26
Pattern comparison sorting speed RT	0.59***	0.21	0.64***	0.51***	-0.36*
Dimensional change card sorting RT	0.69***	0.22	0.81***	*0.63**	-0.44**
Picture sequence memory score	-0.35*	0.04	-0.50***	-0.35*	0.58***

Note: *** =p < .001, **=p < .01, *=p < .05
